# Development and Evaluation of the Online Addiction Medicine Certificate: Free Novel Program in a Canadian Setting

**DOI:** 10.2196/12474

**Published:** 2019-05-24

**Authors:** Lauren Renee Gorfinkel, Amanda Giesler, Huiru Dong, Evan Wood, Nadia Fairbairn, Jan Klimas

**Affiliations:** 1 Mailman School of Public Health Columbia University New York, NY United States; 2 British Columbia Centre on Substance Use Vancouver, BC Canada; 3 School of Population and Public Health University of British Columbia Vancouver, BC Canada; 4 Department of Medicine University of British Columbia Vancouver, BC Canada; 5 School of Medicine University College Dublin Dublin Ireland

**Keywords:** medical education, substance-related disorders, education, distance

## Abstract

**Background:**

Despite the enormous bur­den of disease attributable to drug and alcohol addiction, there remain major challenges in implementing evi­dence-based addiction care and treatment modalities. This is partly because of a persistent lack of accessible, specialized training in addiction medicine. In response, a new online certificate in addiction medicine has been established in Vancouver, Canada, free of charge to participants globally.

**Objective:**

The objective of this study was to evaluate and examine changes in knowledge acquisition among health care professionals before and after the completion of an online certificate in addiction medicine.

**Methods:**

Learners enrolled in a 17-module certificate program and completed pre- and postknowledge tests using online multiple-choice questionnaires. Knowledge acquisition was then evaluated using a repeated measures *t* test of mean test scores before and after the online course. Following the certificate completion, a subset of learners completed the online course evaluation form.

**Results:**

Of the total 6985 participants who registered for the online course between May 15, 2017 and February 22, 2018, 3466 (49.62%) completed the online pretest questionnaire. A total of 1010 participants completed the full course, achieving the required 70% scores. TThe participants self-reported working in a broad range of health-related fields, including nursing (n=371), medicine (n=92), counseling or social work (n=69), community health (n=44), and pharmacy (n=34). The median graduation year was 2010 (n=363, interquartile range 2002-2015). Knowledge of the addiction medicine increased significantly postcertificate (mean difference 28.21; 95% CI 27.32 to 29.10; *P*<.001). Physicians scored significantly higher on the pretest than any other health discipline, whereas the greatest improvement in scores was seen in the counseling professions and community outreach.

**Conclusions:**

This free, online, open-access certificate in addiction medicine appeared to improve knowledge of learners from a variety of disciplines and backgrounds. Scaling up *low threshold* learning opportunities may further advance addiction medicine training, thereby helping to narrow the evidence-to-practice gap.

## Introduction

### Background

Approximately 29 million people are affected by substance use disorders (SUDs) annually [1], with an estimated 21 billion dollars of associated productivity losses in the United States alone [2]. In recent years, a sharp rise in opioid-related deaths has led to numerous public health emergency declarations, and the number of alcohol use disorders is increasing globally [3-5]. Still, uptake of evidence-based SUD treatment is low and people with SUDs often receive inadequate care [6]. This is despite recent progress in addiction science, which has highlighted the important role of skilled health care providers and the efficacy of established psychosocial and pharmacological therapies in improving treatment outcomes [1,6-8]. Moreover, most health care providers frequently report feeling unprepared to effectively identify and treat SUDs, and the stigma toward persons with SUDs persists among the health professions [9-11].

To address these gaps in practice, health professionals need better education and training in addiction medicine. However, such specialized training programs can be inaccessible because of limited space, inability to compensate participants for their time, and the location of training (often big, urban centers) [12-15]. In addition, many of these programs accept only a particular subset of health care professionals—for instance, only physicians or social workers—and have limited resources for expansion. At the same time, effectively training a variety of health care providers in evidence-based treatment of SUDs is critical for providing quality care, as well as curbing the current opioid epidemic. One solution to overcome these challenges is the expansion of online training programs, which can reach a large number of participants, train professionals from a wide range of health disciplines, be delivered at a relatively low cost, and can be accessed from a wide variety of settings [16,17]. Although the literature suggests that online continuing professional development courses in health care can be equally effective as traditional classroom-based courses [17-19], this has not been demonstrated for addiction medicine.

### Objectives

We sought to evaluate changes in knowledge acquisition before and after the completion of a new, comprehensive, and accredited online certificate in addiction medicine, accessible and free of charge to learners worldwide.

## Methods

### Course Description

The Addiction Care and Treatment Online Certificate is a free course and certificate program open for anyone wishing to improve their knowledge of substance use, although it is targeted at health professionals. The course involves 17 modules related to the identification, management, and treatment of SUDs. Textbox 1 lists the course learning objectives and topics covered. Each module is comprised a 20- to 75-min video lecture (including a slideshow with spoken voice-over) by an expert in the relevant topic. Each module (with the exception of modules 1 and 17) is then followed by a brief multiple-choice test of the module material. All multiple-choice questions are in single best answer format with 4 answer options. Those looking to obtain a formal certificate and Continuing Medical Education certificate must complete all the modules, achieving a minimum passing score of 70% on all postmodule tests. Overall, the online course takes approximately 16 hours to complete; however, it is self-paced, so learners can complete each module in their own time. Modules do not have to be completed in chronological order, allowing learners to select the topics that most interest or benefit them. Once learners complete the course, they must pass through an online evaluation form (with one mandatory question on potential bias in course content) to receive a digital certificate.

Before release, this course was accredited via review and approval by the University of British Columbia Division of Continuing Professional Development. This required a formal committee with representation of the target audience, a high degree of evidence, and no industry bias or involvement. The course also met the certification criteria of the College of Family Physicians of Canada, as well as Maintenance of Certification Program of The Royal College of Physicians and Surgeons of Canada.

### Course Development and Background

On the basis of the informal and formal consultations with a range of stakeholders by the course’s planning committee, it was clear that there was a general lack of awareness of the range of evidence-based treatments for SUDs. Specifically, there was an unmet need for specific education and training for health care professionals on SUDs that was accessible and provided at no cost. In response to this need, the British Columbia Centre on Substance Use (BCCSU) developed this structured course in partnership with content experts throughout British Columbia. This included physicians in the areas of psychiatry, public health, internal medicine, and family medicine. Although the target audience of the program is primarily prescribers, modules were created to be accessible to all allied health disciplines, including nurses, social workers, psychologists, and were also accessible for a general audience.

The course design utilizes a case-based learning structure, with theoretical and academic context nested in the introductions to the module. The topics, content, and speakers were selected through recommendations and advice from the planning committee. Each module is taught by a faculty member who has extensive experience with the particular topic and would be considered an expert in the province. Physician members of the planning committee were instrumental in ensuring that all materials were evidence-based and relevant to physicians practicing in the primary care environments. Before beginning their presentations, the course’s lead author (EW) and the planning committee required faculty members to submit detailed module outlines. These were thoroughly reviewed, and feedback was returned to presenters for incorporation into their lecture and presentation slides. The finalized presentations were then reviewed to ensure validity and objectivity of content. The lectures were recorded between August 2015 to May 2016. The course was hosted on the host center’s website using a WordPress content management system.

**Learning objectives and topics covered in The Addiction Care and Treatment Online Certificate.**

Learning objectives

1. Incorporating screening diagnosis and brief intervention and referral to treatment for substance use disorders in clinical practice.
2. Selecting the appropriate pharmacological and psychosocial treatment interventions based on the best evidence, as well as individual patient needs, circumstances, and preferences.
3. Providing safe and effective treatment to patients and their families throughout the induction, maintenance, and/or discontinuation process across the continuum of care for substance use disorders.
4. Setting treatment goal monitoring and evaluating progress and providing patient-centered support across the continuum of care for substance use disorders.
5. Appreciating the complexity of substance use disorders, diversity of care, and providing informed referrals to evidence-based support services.
6. Promoting recovery, safety, wellness, and harm reduction to improve patient care and support for those with substance use disorders.
7. Implementing strategies for safer prescribing practices for medications with abuse/diversion potent (ie, opioids for analgesia, benzodiazepines, etc).

Topics or modules (time in module)

1. Introduction to addiction medicine (20 min)
2. Screening, diagnosis, and brief intervention for substance use disorders (45 min)
3. Opioid use disorder (50 min)
4. Tobacco use disorder (45 min)
5. Alcohol use disorder (35 min)
6. Withdrawal syndromes (60 min)
7. Stimulant use disorder (20 min)
8. Polysubstance use (65 min)
9. Comorbid mental illness and substance use disorders (35 min)
10. Pain and substance use disorders (65 min)
11. Common medical complications (45 min)
12. Safe prescribing (75 min)
13. Overdose prevention and harm reduction (45 min)
14. Psychosocial interventions (50 min)
15. Addiction in the workplace (75 min)
16. Recovery oriented systems of care (30 min)
17. Cases consolidating knowledge (35 min)

The course leads and advisory committee guide the ongoing, year-round recruitment activities. Participant recruitment strategies include disseminating advertisements through electronic mailing lists, posters, brochures, descriptions and links on the bccsu.ca website, lay media advertisements, conference participation, newsletters, social media, and word of mouth. In addition, the course was promoted through in-person seminars focused on substance use across Canada.

### Procedures

To take the online course, participants first had to register using the online registration form. Here, learners were given space to fill in their full name and email address and were asked to select their home province from a drop-down menu (all Canadian provinces and *Other* were listed). Providing province information became mandatory after the first month of the course. In the registration form, learners were also asked to select their professional discipline from a given list. Following registration, learners completed a multiple-choice knowledge test (the *pretest*) to evaluate baseline knowledge of course content. In addition, following each module, learners completed multiple-choice knowledge tests (the *posttests*) to evaluate the understanding of the material just taught.

As 2 of the modules (1 and 17, the introduction and conclusion) lacked posttests, to pass the online course, learners had to complete a total of 15 posttests. When all posttests were completed with a minimum 70% score, learners were then given the option to complete an online evaluation form, gauging their satisfaction with the program and the applicability of course material to their clinical practice. The evaluation form also asked for further demographic information, such as professional discipline, the year that learners completed their professional degrees, and the health care settings in which they provide services.

**Sample question and format.**

Question: Evidence-based first-line anticraving and relapse prevention therapies for the treatment of alcohol use disorder include: (1) Naltrexone 50 mg once daily; (2) Acamprosate 666 mg once daily; (3) Gabapentin 300 mg once daily; (4) Celexa 40 mg once daily.

Correct answer: 1

Explanation: Naltrexone is typically provided 50 mg once daily and has a number needed to treat to prevent a return to any drinking of 20. Acamprosate has a number needed to treat to prevent a return to any drinking of 12 and is an alternative first-line agent, but it is dosed 666 mg 3 times per day rather than once daily. Though less studied, Gabapentin appears to be an effective anticraving agent but the optimal studied dose was 600 mg 3 times per day. Celexa is not a pharmacotherapy for alcohol use disorder.

Citations:

1. Jonas DE et al. Pharmacotherapy for adults with alcohol use disorders in outpatient settings: a systematic review and meta-analysis. *JAMA*. 2014 May 14;311(18):1889-900.
2. Mason BJ et al. Gabapentin treatment for alcohol dependence: a randomized clinical trial. *JAMA Intern Med*. 2014 Jan;174(1):70-7.

### Survey Development

Questions on pre- and posttests were collaboratively developed by the course’s lead author (EW) and the lecturers. Questions were updated by the course coordinator (AG), BCCSU staff, and experienced clinicians in addiction medicine. All pre- and posttests used a multiple-choice format and were designed to measure changes in learners’ knowledge of addiction medicine and SUDs. The pretest contains 30 knowledge questions of material from all course modules. Each posttest contains 3 to 10 multiple-choice questions, covering only material from the relevant module. Textbox 2 represents the question format adhered to in this course. Although all pretest questions were asked postcourse, it was impossible for these questions to be matched item-to-item with the pretest results, because of the online platform setup. Although a minimum score of 70% was required to pass each posttest, there was no such requirement for the 30-item pretest. Learners can also attempt posttests multiple times, resulting in the number of learners passing each module being unequal to the number of all recorded attempts at each posttest. As a result of learner feedback, some posttest questions were altered over the course of the study period. For example, changes were made to the phrasing of questions in the module *Alcohol Use Disorder*.

This study was approved by the Research Ethics Board at Providence Health Care Research Institute, University of British Columbia. All participants were informed of the study purpose, as well as the voluntary and anonymous nature of participation before signing the informed electronic consent.

### Data Analysis

Data from the course registration and pre- and posttests were linked using participants’ full names and email addresses. Using registration data, participants were divided into 7 broad health-related fields: (1) *medicine*, (2) *nursing*, (3) *pharmacy*, (4) *counseling/social work*, (5) *community outreach/support work*, (6) *residents/students*, and (7) *other*.

We measured the effectiveness of the course using (1) completion rate (percentage of participants who attempted all 15 posttests out of the total number of participants registered), (2) success rate (percentage of participants who successfully passed all 15 posttests out of the total number of participants who attempted *all* 15 posttests), (3) commitment rate (percentage of participants who passed all 15 posttests out of the total number of participants who attempted at least *one* posttest), and (4) mean difference between the assessments at the start (pretest) and at the end (posttests) of the online course. The pretest score was used as a proxy for precourse knowledge and the mean of all posttest scores was used as an indicator of the overall postcourse knowledge. A repeated measures design with *t* tests of mean scores on pre- and posttests therefore evaluated positive knowledge acquisition in participants who completed all 15 posttests. Scores on all tests were measured in percentage terms. When a participant attempted a posttest more than once, the mean score from all *attempts* by that participant was taken (not only those which surpassed 70%), so as to keep one posttest value per participant and module.

We also examined differences in test scores between participants in different health-related fields (eg, nursing, pharmacy). Health professionals with significantly greater pretest scores were taken to have greater baseline knowledge than other health professionals taking the course. In addition, health professionals with a significantly greater difference in scores were taken to have had greater knowledge benefits than other health professionals taking the course. Linear regression and paired *t* tests were used to test statistical significance of these differences with SAS 9.4 (SAS Institute). All *P* values were 2-sided.

## Results

### Registration and Participant Characteristics

**Figure 1:**
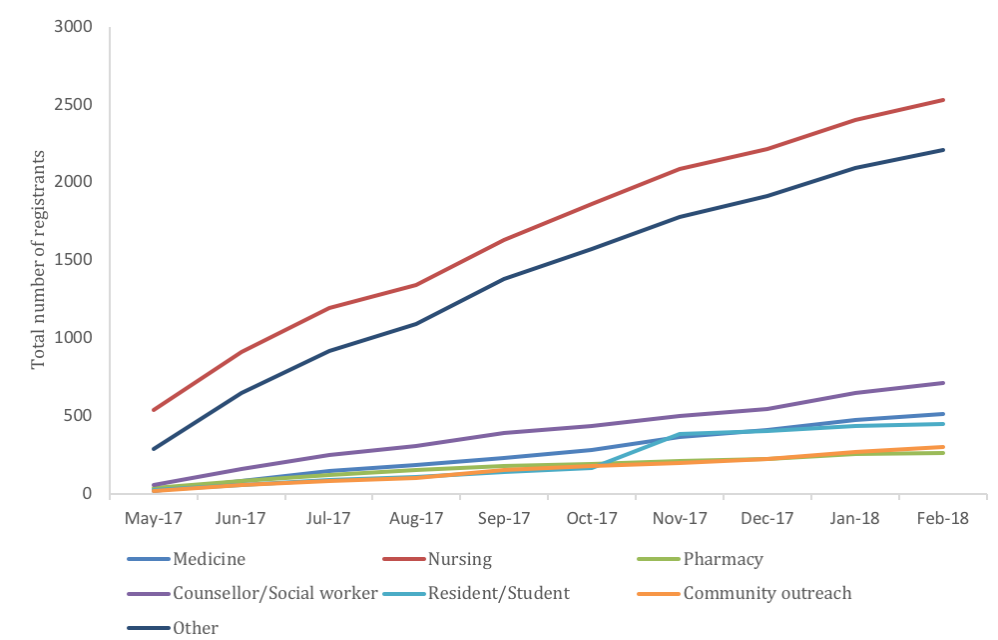
Total number of registrations in the online addiction medicine certificate from May 2017 to February 2018, stratified by professional discipline (N=6985). Jan: January; Feb: February; Jun: June; Jul: July; Aug: August; Sep: September; Oct: October; Nov: November; Dec: December.

Between May 15, 2017 and February 22, 2018, a total of 6985 persons registered for the course. During this period, there was a steady linear increase in the total number of course registrations, with a particularly sharp rise in the number of nurses (Figure 1). Of those who registered, 3466 completed the pretest and attempted at least one module’s posttest. A total of 1010 then attempted every posttest at least once, all of whom achieved the minimum passing score of 70% on one attempt of each test. Therefore, the course had a completion rate of 14.45%, a success rate of 100%, and a commitment rate of 29.14%.

Among the 1010 course completers, participants self-reported working in a broad range of health-related fields, mostly nursing (371/1010, 36.73%) and medicine (92/1010, 9.11%; Table 1). There was an overall difference in the province of origin between those who did and did not complete the course (*P*=.013), with a higher proportion of Ontarians and Prairie residents among completers versus noncompleters (Table 1).

### Knowledge of Addiction Medicine

Attempting (regardless of pass/fail status) all 15 modules with posttests was a study inclusion criterion. However, as all learners who attempted every module also passed the course, a total of 1010 *attempters* were included in the analyses.

Postcertificate, the knowledge of addiction medicine increased significantly (mean difference 28.2; 95% CI 27.3 to 29.1; *P*<.001; Table 2). Physicians scored between 6.9 and 10.6 percentage points higher on the pretest than other health disciplines. The greatest improvement in scores was seen in the counseling and community outreach professions (Table 3).

**Table 1:** Sociodemographic characteristics of the sample, with *P* values, stratified by certificate completion status.

Characteristic			Total (N=3466), n (%)		Completion status, n (%)				*P* value
					Completers (n=1010)		Ongoing learners^a^ (n=2456)		
**Province**									.013
	British Columbia	1371 (39.56)		362 (35.84)		1009 (41.08)			
	Prairie provinces (SK, MB, AB)^b^	716 (20.66)		237 (23.47)		479 (19.50)			
	Ontario	237 (6.84)		71 (7.03)		166 (6.76)			
	Quebec/Atlantic regions (NB, NL, NS, PEI)^c^	101 (2.91)		24 (2.38)		77 (3.14)			
	Missing/other^d^	1041 (30.03)		316 (31.29)		725 (29.52)			
**Self-identified discipline**									<.001
	Medicine	274 (7.91)		92 (9.11)		182 (7.41)			
	Nursing	1265 (36.50)		371 (36.73)		894 (36.40)			
	Counseling or social work	346 (9.98)		69 (6.83)		277 (11.28)			
	Pharmacy	124 (3.58)		34 (3.37)		90 (3.66)			
	Student or resident	239 (6.90)		61 (6.04)		178 (7.25)			
	Community outreach	167 (4.82)		44 (4.36)		123 (5.01)			
	Other	1051 (30.32)		339 (33.56)		712 (28.99)			
**Practice setting^e^** **(n=475)**									—^f^
	Community-based organization	—		99 (17.3)		—			
	Physician office–based practice	—		64 (11.2)		—			
	Private drug treatment clinic	—		18 (3.1)		—			
	Provincial health authority	—		152 (26.6)		—			
	Community health center	—		101 (17.7)		—			
	Other^g^	—		138 (24.1)		—			

^a^This includes participants who may have registered but have not yet started the course, participants who had not yet completed the course at the time of data collection, and participants who completed the course but did not pass.

^b^SK: Saskatchewan; MB: Manitoba; AB: Alberta.

^c^NB: New Brunswick; NL: Newfoundland; NS: Nova Scotia; PEI: Prince Edward Island.

^d^This includes participants from international settings, as well as those with missing location data.

^e^Only participants who passed the course filled in this information in the satisfaction form; totals do not add up to 475 as participants could select more than one service setting.

^f^Not applicable.

^g^Other settings included hospitals, mental health facilities, pharmacies, group homes, and more.

**Table 2:** Knowledge in addiction medicine among learners completing the free online certificate (n=1010).

Module			Statistics
Pretest total, mean percentage score (SD)			52.6 (14.5)
**Posttest scores per module, mean percentage score (SD)**			
	Screening, diagnosis, and brief interventions	84.9 (14.3)	
	Opioid use disorder	78.1 (21.7)	
	Tobacco use disorder	88.3 (15.4)	
	Alcohol use disorder	75.1 (23.2)	
	Withdrawal syndromes	79.6 (21.9)	
	Stimulant use disorder	76.5 (19.1)	
	Polysubstance use	75.1 (26.5)	
	Comorbid mental illness and substance use disorder	77.1 (24.8)	
	Pain and substance use disorders	77.8 (22.6)	
	Common medical complications	78.1 (21.9)	
	Safe prescribing	74.1 (24.3)	
	Overdose prevention and harm reduction	83.5 (22.0)	
	Psychosocial interventions	81.9 (18.0)	
	Addiction in the workplace	85.5 (17.5)	
	Recovery-oriented systems of care	81.4 (18.0)	
Posttest total, mean percentage score (SD)			80.8 (8.7)
Mean difference between total pre- and posttest scores (95% CI)			28.2^a^ (27.3-29.1)

^a^*P*<.001.

**Table 3:** Pairwise comparisons between medicine and other disciplines on pretest scores and change in score from pre- to posttest (n=1010).

Discipline	Pretest scores, mean difference (95% CI)^a^	Change in pre- and posttest scores, mean difference (95% CI)^a^
Medicine	Reference group	Reference group
Nursing	−8.4^b^ (−14.1 to −2.8)	2.6 (−0.7 to 5.8)
Pharmacy	−8.2^c^ (−12.8 to −3.6)	5.0 (−0.7 to 10.6)
Resident/student	−7.9^b^ (−11.1 to −4.6)	3.3 (−1.4 to 7.9)
Counseling/social work	−9.9^b^ (−14.3 to −5.4)	5.6^d^ (1.1 to 10.0)
Community outreach/support worker	−10.6^b^ (−15.8 to −5.5)	7.9^e^ (2.7 to 13.0)
Other	−6.9^d^ (−10.2 to −3.6)	2.3 (−1.0 to 5.6)

^a^ Negative mean difference signifies a score/change in scores lower than that of the reference group. Positive mean difference signifies a score/change in scores higher than that of the reference group.

^b^*P*<.001.

^c^*P*=.003.

^d^*P*=.015.

^e^*P*=.003.

A subset of 475 participants (47.0%) completed the course satisfaction form. The most commonly reported service settings were provincial health authorities, community health centers, and community-based organizations (Figure 1). The median graduation year was 2010 (n=363, interquartile range 2002-2015). Most (89%) of the participants who completed the course evaluation either agreed or strongly agreed that the course successfully met their learning needs. In addition, the majority of the participants rated the course’s relevance to their practice (83%) and incorporation of evidence-based research (93%) as *above average* or *excellent* (Table 4).

**Table 4:** Participant responses to select evaluation criteria for the Addiction Care and Treatment Online Certificate.

Statement	Rating (%)					
	Strongly disagree / Poor	Disagree / Below average	Neutral / Average	Agree / Above average	Strongly agree / Excellent	Not applicable
This course was effective in meeting my learning needs	2.5	1.7	5.3	33.3	54.5	2.7
Relevance to my practice	0.2	1.9	12.6	38.9	43.3	3
Incorporation of evidence-based research	0.2	0.2	4.8	33.2	57.9	3.6

## Discussion

### Principal Findings

We sought to evaluate the changes in knowledge acquisition before and after completion of a comprehensive online certificate in addiction medicine. Following the course, the knowledge of addiction medicine increased significantly, with a completion rate of 14.5% (percentage of participants who attempted all 15 posttests out of the total number of participants registered), success rate of 100% (percentage of participants who successfully passed all 15 posttests out of the total number of participants who attempted *all* 15 postests), and commitment rate of 29.1% (percentage of participants who passed all 15 posttests out of the total number of participants who attempted at least *one* posttest). Physicians scored significantly higher on the pretest than any other health discipline, whereas the greatest improvement in scores was seen in the counseling professions and community outreach. A majority of participants reported that the course was effective in meeting their learning needs, was relevant to their practice, and well-incorporated evidence-based research.

This course is a novel online training program in addiction medicine—a field in urgent need of expanded educational opportunities for health care providers [9,10,20]. As of February 2018, the course had nearly 7000 registrants, confirming the strength of low threshold, online models for facilitating rapid scale-up of evidence-informed training in addiction medicine, and a high demand [21-23]. This demand was particularly notable among nurses who composed the largest proportion of health providers in our sample and saw the sharpest increase in registrations. As reported in previous papers [24], the completion rates of open online courses are often lower than those of traditional in-person training, and this may be more a product of participants’ individual preferences or needs, rather than the course material or structure. As such, the observed completion rate of this course is in line with previous studies of open online courses, which report completion rates from 0.9% to 36.1% (median 6.5%) [24,25]. It is also important to note that the online certificate in addiction medicine was intentionally structured for participants to select the modules most relevant to them.

Similar to previous studies of online courses on SUDs, we found a significant increase in knowledge of addiction medicine postcertificate [22,23,26,27]. In addition, this study highlighted which health professions may derive the most benefit from such a course. Aligned with prior literature, which has shown that education in SUD care is often lacking in social work and counseling curricula [22,28], participants in counseling/social work and community outreach demonstrated the greatest improvement in scores. For example, in one study of university-level counseling and social work programs, it was found that just 69% of masters-level counseling programs, 3% of bachelors-level social work programs, and 2% of masters-level social work programs required a course in SUD care [29]. Still, the online format can present unique barriers—including time- and schedule-constraints—as noted by previous studies of online training in SUDs [30].

### Limitations

Several limitations may reduce the generalizability of our findings. First, because of the inconsistencies in the data, for some participants, it was impossible to link their course registration data and test scores. Second, the large number of participants from western Canada may have introduced bias into the results, as health care professionals’ knowledge and training in addiction medicine may vary by setting. Promoting the course to a more international audience could improve training and highlight the needs of health care professionals in a wider range of contexts. Third, the self-selection of registrants for the course may mean that the study participants were more likely to have a higher level of interest or experience in addiction medicine––it is likely that practitioners who seek specialized training are more prone to positive attitudes toward, and learning experiences with people who have SUDs [31]. Fourth, as participants were able to attempt each postmodule test as many times as they liked, and a minimum score of 70% was required to pass each test, our overall posttest value may have been positively skewed. This limitation was carefully considered before analyses, balancing perspectives that a participant’s final passing attempt at each posttest (1) represented new retained knowledge and (2) was the product of selecting the correct answer by chance. Therefore, we averaged the scores from all attempts by a single participant at each posttest. This mean score was then used to calculate a total group mean score for the course. Finally, we did not capture a corresponding change in provider behavior following the course. Future research examining the impact of this course in addiction care settings would be valuable.

### Conclusions

In this study, over 6000 participants began and over 1000 participants completed online training in addiction medicine. Overall, our analyses suggest that the course can feasibly increase knowledge in addiction medicine to a wide range of health care providers. Scaling up *low threshold* learning opportunities may further advance addiction medicine training, thereby helping to narrow the evidence-to-practice gap.

## Abbreviations

BCCSU: British Columbia Centre on Substance Use
SUD: substance use disorder
